# Mitochondrial apoptosis imaged by high-throughput confocal microscopy

**DOI:** 10.1038/s41597-026-08502-7

**Published:** 2026-10-15

**Authors:** Lina Hellwig, Felix J. Meigel, Harald Stachelscheid, Steffen Rulands, Philipp Mergenthaler

**Affiliations:** 1 Charité – University Medical Center Berlin, Center for Stroke Research Berlin, Berlin, Germany; 2 Charité – University Medical Center Berlin, Department of Neurology with Experimental Neurology, Berlin, Germany; 3 Ludwig-Maximilians-Universität München, Arnold Sommerfeld Center for Theoretical Physics and Center for Nanoscience, Theresienstr. 37, 80333 München, Germany; 4 Berlin Institute of Health at Charité – University Medical Center Berlin, Core Unit pluripotent Stem Cells and Organoids, Berlin, Germany; 5 Radcliffe Department of Medicine, University of Oxford, Oxford, UK

## Abstract

Mitochondrial programmed cell death (i.e. intrinsic apoptosis) is regulated through the formation of pro-apoptotic protein complexes at the mitochondrial outer membrane (MOM), which permeabilize the MOM and commit the cell to die. This step is modulated by mitochondrial dynamics. To capture this, we treated cells with the mitochondrial dynamics inhibitor M1 before inducing apoptosis with actinomycin D (ActD), and imaged them by high-throughput spinning-disk confocal microscopy. In mouse embryonic fibroblasts (MEFs) we followed apoptosis by long-term live-cell imaging, and in human induced pluripotent stem cells (hiPSCs) we stained fixed cells for the pro-apoptotc protein BAX at mitochondria. Here, we provide a description of these two large-scale microscopy datasets. Dataset 1 contains 637,632 images of MEFs imaged as time series after labelling of mitochondrial morphology and integrity with the mitochondrial membrane potential-sensitive dye tetramethylrhodamine-ethylester (TMRE) and apoptosis with Annexin V-FITC. The dataset also contains a digital phase contrast channel which can be useful to track cells. Dataset 2 contains 9,504 images of hiPSCs with immunofluorescence labelling of BAX and COX IV. The datasets are made available through the BioImage Archive repository.

## Background

Since its discovery in 1972, apoptosis — the process of programmed cell death — has been a central focus of research across many biological and biomedical disciplines^1,2^. Apoptosis is a fundamental biological mechanism in development^1^ and disease, including cancer^3^ and acute neurodegeneration such as in stroke^4,5^. Two main signalling routes are known to trigger and execute apoptosis: the extrinsic and the intrinsic apoptotic pathways^1^. The extrinsic pathway is initiated by the binding of specific ligands to death receptors on the cell surface, thereby activating a cascade of downstream signalling events^1^. The intrinsic pathway is regulated by intracellular stress or damage signals that activate pro-apoptotic proteins within the cell and ultimately signal through mitochondria (i.e., mitochondrial apoptosis)^1^. These events converge on the mitochondrial outer membrane (MOM), whose permeabilization (MOMP) marks a critical step in the apoptotic process^6,7^. A key mediator of this step is the pro-apoptotic Bcl-2 family member BAX^6^. In its inactive form, BAX resides in the cytosol and translocates to the MOM upon activation through cell death-promoting signals, where it undergoes conformational changes and assembles into oligomeric complexes in the MOM^6,7^. These complexes form pores in the MOM, leading to the release of cytochrome c into the cytosol, which is the generally irreversible step that drives the cell towards execution of apoptosis and ultimately cellular disintegration^1,6^. The temporal kinetics of apoptosis and the individual steps leading to MOMP, cytochrome c release, caspase activation, loss of cell membrane integrity and cellular disintegration can vary substantially, depending on cell type, apoptotic stimulus, and even between individual cells in the same culture^8–12^. The latency to reach apoptotic commitment and the accumulation of apoptotic cells in a population can occur over minutes to many hours or even days in a cell-type- and stimulus-dependent manner^13–17^. However, in certain circumstances, MOMP or cytochrome c release from mitochondria does not necessarily commit cells to undergo apoptosis^18–20^.

Processes as critical as apoptosis have to be tightly regulated and mechanisms need to be in place for cells to distinguish relevant from irrelevant stress signals. Mitochondria play a central role in this regulation^21^. We addressed this regulation in the study underlying the datasets described here^21^. We show that mitochondria, which as highly dynamic cell organelles continuously undergo rapid fission and fusion^22^, thereby change the concentration of BAX complexes in the MOM and influence the cell’s decision to undergo apoptosis^21^. Accordingly, the readouts used in our study^21^ are BAX localization (i.e., BAX immunofluorescence) and execution-phase apoptosis (i.e., Annexin V staining), rather than the kinetics of cytochrome c release or MOMP.

The two datasets described herein address apoptosis in cellular systems in the context of mitochondrial dynamics, each designed to capture complementary aspects of the apoptotic process. Dataset 1 provides time-resolved information on the onset of the execution phase of apoptosis in immortalized mouse embryonic fibroblasts (MEFs), with Annexin V serving as a quantifiable readout of execution-phase apoptosis alongside simultaneous assessment of mitochondrial structure, and mitochondrial membrane potential via the potential-sensitive dye tetramethylrhodamine-ethylester (TMRE). Dataset 2 complements this by providing spatially resolved immunofluorescence data on BAX recruitment to mitochondria in human induced pluripotent stem cells (hiPSCs), offering insight into the immediate execution step of mitochondrial apoptosis in the context of altered mitochondrial dynamics.

**Fig. 1:**
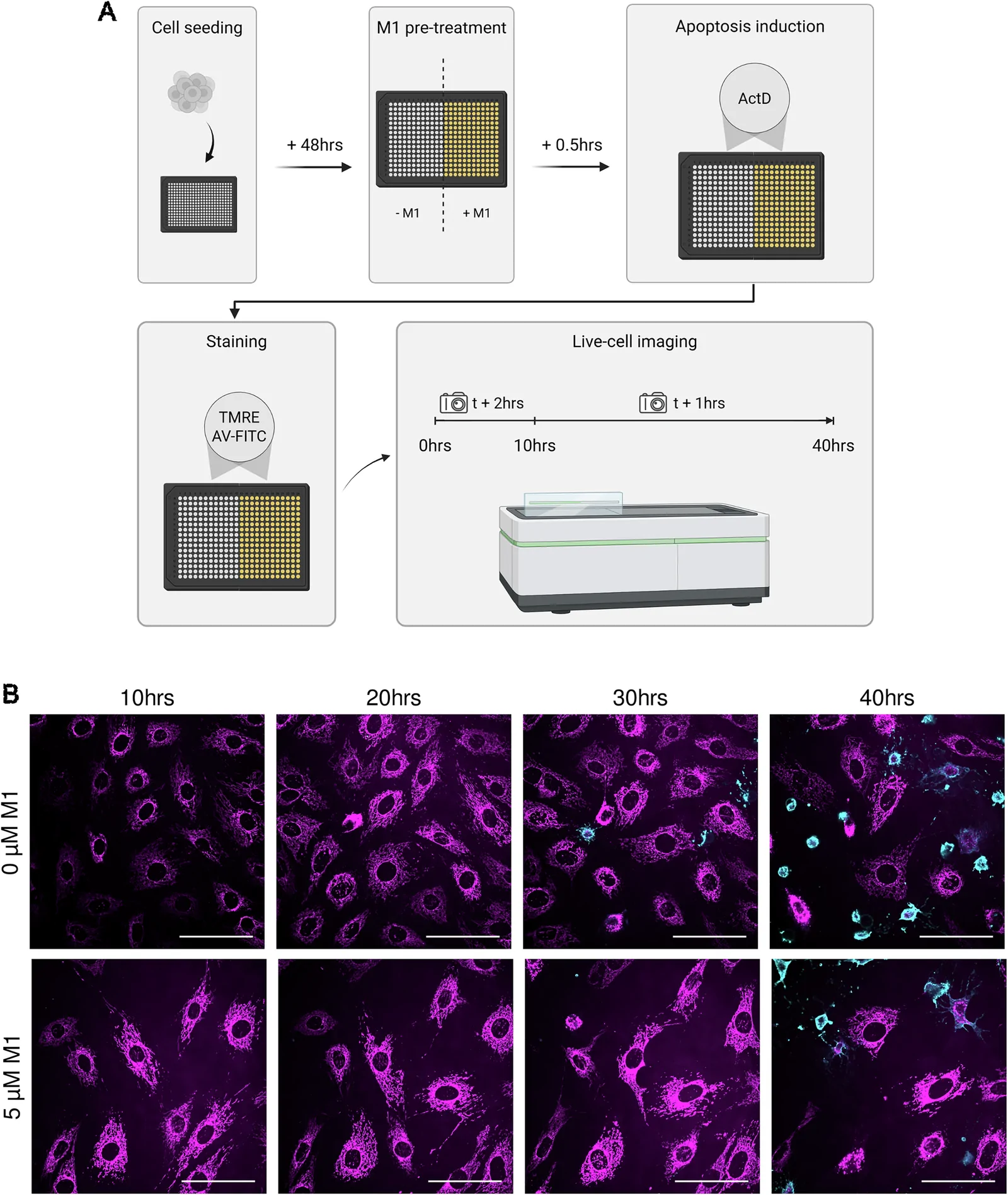
(**A**) Schematic overview of the experimental setup. Briefly, 48 hours after seeding, MEFs were incubated with M1 (“ + M1”) or vehicle (DMSO, “-M1”), and after 30 minutes treated with ActD, stained with TMRE and Annexin V-FITC (“AV-FITC”), and subsequently imaged for 40 hours (see text for details). (**B**) Confocal images of MEFs, here treated with 120 nM ActD for apoptosis induction at the indicated time points. Cells were imaged consecutively in the same field of view. Cyan: Annexin V, magenta: TMRE. Scale bars: 100 µm. *Panel A was created with BioRender.com*.

**Fig. 2:**
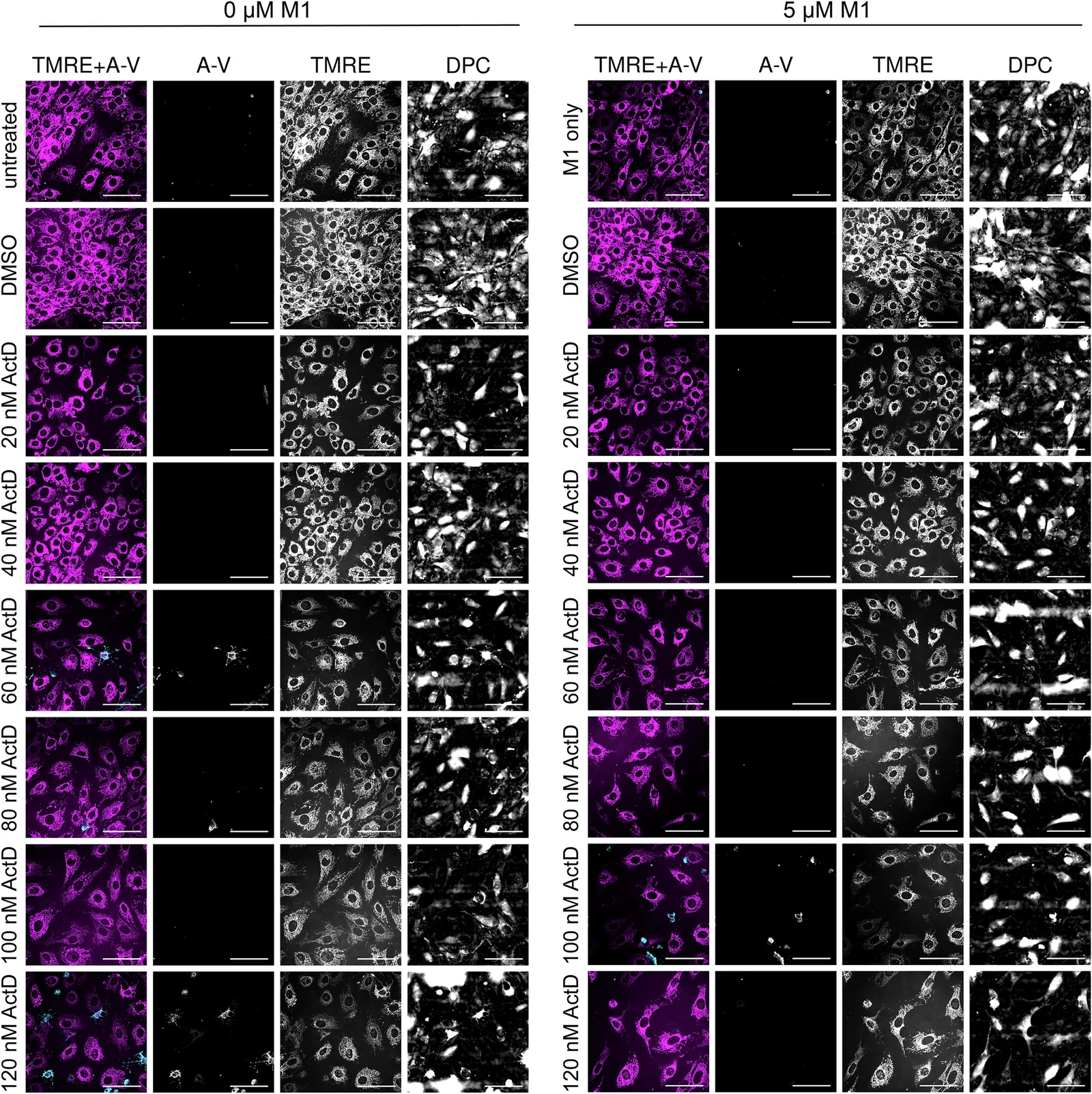
Confocal images of MEFs 30 h after treatment with indicated concentrations of ActD for apoptosis induction. DMSO was applied as control (i.e., 0 nM ActD). 30 mins before apoptosis induction, cells were either pre-treated with M1 (“5 µM M1”) or vehicle (DMSO, “0 µM M1”). A-V: Annexin V; scale bars: 100 µm.

Dataset 1 (Figs. 1,2) was used to investigate how mitochondrial dynamics influence apoptotic cell-fate decisions^21^. For induction of mitochondrial apoptosis, we chose actinomycin D (ActD), which triggers BAX activation^15^, and the compound hydrazone M1, which has been described as a mitochondrial fusion promoter^23^, but which in our system functions more broadly as a mitochondrial dynamics inhibitor^21^. MEFs were selected for their well-characterized mitochondrial morphology and as the cell line used in the original characterization of M1 (Ref. ^23^). We performed live-cell high-content confocal imaging in MEFs pre-treated with M1 or with DMSO as vehicle control for M1 and subsequently exposed to increasing concentrations of ActD in DMSO (Figs. 1,2, Table 1). During the first 10 h of imaging, images for all channels were acquired every two hours. For the remaining time, images were acquired every hour, enabling the visualization of mitochondrial dynamics and execution-phase apoptosis in individual cells over a total timeframe of 40 hours (Fig. 1). Mitochondrial morphology and integrity were monitored using TMRE. Apoptosis was detected with Annexin V-FITC, which binds to phosphatidylserine exposed on the outer leaflet of the plasma membrane during the execution phase of apoptosis. The dataset also contains a digital phase contrast (DPC) channel, which serves as a computationally reconstructed contrast method that can be useful to track cells without the need for cell labels. This method however is prone to background artefacts, and reconstruction quality may vary depending on focus stability and acquisition settings, which should be considered when using this channel for label-free segmentation or tracking applications. In this specific dataset, DPC image quality was dependent on cell density and was not used in our analyses^21^. Example images can be found in Fig. 2. To our knowledge, no publicly available dataset currently combines apoptotic markers with both unaffected and perturbed mitochondrial dynamics, making this resource useful for interdisciplinary research between mitochondrial biology, cell death signalling, and computational image analysis.

**Table 1:** Overview Live-Cell Imaging of MEFs (Dataset 1).

Imaging Set	Replicate ID	Cell Type	Treatment condition	ActD Concentration [nM]	M1 Concentration [µM]	No. of Wells	No. of Fields per Well	No. of Timepoints	No. of Planes	Channels	Objective	Image Size	Pixel Size [µm]	Bit Depth	Total Image Count
Live-Cell Imaging Set1	N1	MEF	Untreated	0	0	3	12	36	1	DPC, TMRE, AV-FITC	40x Water, NA = 1.1	2160x2160	0.1483	16-bit	46,656
			DMSO	0	0	3	12								
			ActD	20, 40, 60, 80	0	12	12								
			M1	0	5	3	12								
			M1+DMSO	0	5	3	12								
			M1+ActD	20, 40, 60, 80	5	12	12								
Live-Cell Imaging Set2	N2	MEF	Untreated	0	0	3	12	36	1	DPC, TMRE, AV-FITC	40x Water, NA = 1.1	2160x2160	0.1483	16-bit	93,312
			DMSO	0	0	3	12								
			ActD	20, 40, 60, 80	0	12	12								
			M1	0	5	3	12								
			M1+DMSO	0	5	3	12								
			M1+ActD	20, 40, 60, 80	5	12	12								
	N3	MEF	Untreated	0	0	3	12	36	1	DPC, TMRE, AV-FITC	40x Water, NA = 1.1	2160x2160	0.1483	16-bit	
			DMSO	0	0	3	12								
			ActD	20, 40, 60, 80	0	12	12								
			M1	0	5	3	12								
			M1+DMSO	0	5	3	12								
			M1+ActD	20, 40, 60, 80	5	12	12								
Live-Cell Imaging Set3	N4	MEF	Untreated	0	0	3	12	36	1	DPC, TMRE, AV-FITC	40x Water, NA = 1.1	2160x2160	0.1483	16-bit	1,24,416
			DMSO	0	0	3	12								
			ActD	20, 40, 60, 80, 100, 120	0	18	12								
			M1	0	5	3	12								
			M1+DMSO	0	5	3	12								
			M1+ActD	20, 40, 60, 80, 100, 120	5	18	12								
	N5	MEF	Untreated	0	0	3	12	36	1	DPC, TMRE, AV-FITC	40x Water, NA = 1.1	2160x2160	0.1483	16-bit	
			DMSO	0	0	3	12								
			ActD	20, 40, 60, 80, 100, 120	0	18	12								
			M1	0	5	3	12								
			M1+DMSO	0	5	3	12								
			M1+ActD	20, 40, 60, 80, 100, 120	5	18	12								
Live-Cell Imaging Set4	N6	MEF	Untreated	0	0	3	12	36	1	DPC, TMRE, AV-FITC	40x Water, NA = 1.1	2160x2160	0.1483	16-bit	1,24,416
			DMSO	0	0	3	12								
			ActD	20, 40, 60, 80, 100, 120	0	18	12								
			M1	0	5	3	12								
			M1+DMSO	0	5	3	12								
			M1+ActD	20, 40, 60, 80, 100, 120	5	18	12								
	N7	MEF	Untreated	0	0	3	12	36	1	DPC, TMRE, AV-FITC	40x Water, NA = 1.1	2160x2160	0.1483	16-bit	
			DMSO	0	0	3	12								
			ActD	20, 40, 60, 80, 100, 120	0	18	12								
			M1	0	5	3	12								
			M1+DMSO	0	5	3	12								
			M1+ActD	20, 40, 60, 80, 100, 120	5	18	12								
Live-Cell Imaging Set5	N8	MEF	Untreated	0	0	3	12	36	1	DPC, TMRE, AV-FITC	40x Water, NA = 1.1	2160x2160	0.1483	16-bit	1,24,416
			DMSO	0	0	3	12								
			ActD	20, 40, 60, 80, 100, 120	0	18	12								
			M1	0	5	3	12								
			M1+DMSO	0	5	3	12								
			M1+ActD	20, 40, 60, 80, 100, 120	5	18	12								
	N9	MEF	Untreated	0	0	3	12	36	1	DPC, TMRE, AV-FITC	40x Water, NA = 1.1	2160x2160	0.1483	16-bit	
			DMSO	0	0	3	12								
			ActD	20, 40, 60, 80, 100, 120	0	18	12								
			M1	0	5	3	12								
			M1+DMSO	0	5	3	12								
			M1+ActD	20, 40, 60, 80, 100, 120	5	18	12								
Live-Cell Imaging Set6	N10	MEF	Untreated	0	0	3	12	36	1	DPC, TMRE, AV-FITC	40x Water, NA = 1.1	2160x2160	0.1483	16-bit	1,24,416
			DMSO	0	0	3	12								
			ActD	20, 40, 60, 80, 100, 120	0	18	12								
			M1	0	5	3	12								
			M1+DMSO	0	5	3	12								
			M1+ActD	20, 40, 60, 80, 100, 120	5	18	12								
	N11	MEF	Untreated	0	0	3	12	36	1	DPC, TMRE, AV-FITC	40x Water, NA = 1.1	2160x2160	0.1483	16-bit	
			DMSO	0	0	3	12								
			ActD	20, 40, 60, 80, 100, 120	0	18	12								
			M1	0	5	3	12								
			M1+DMSO	0	5	3	12								
			M1+ActD	20, 40, 60, 80, 100, 120	5	18	12								

**Fig. 3:**
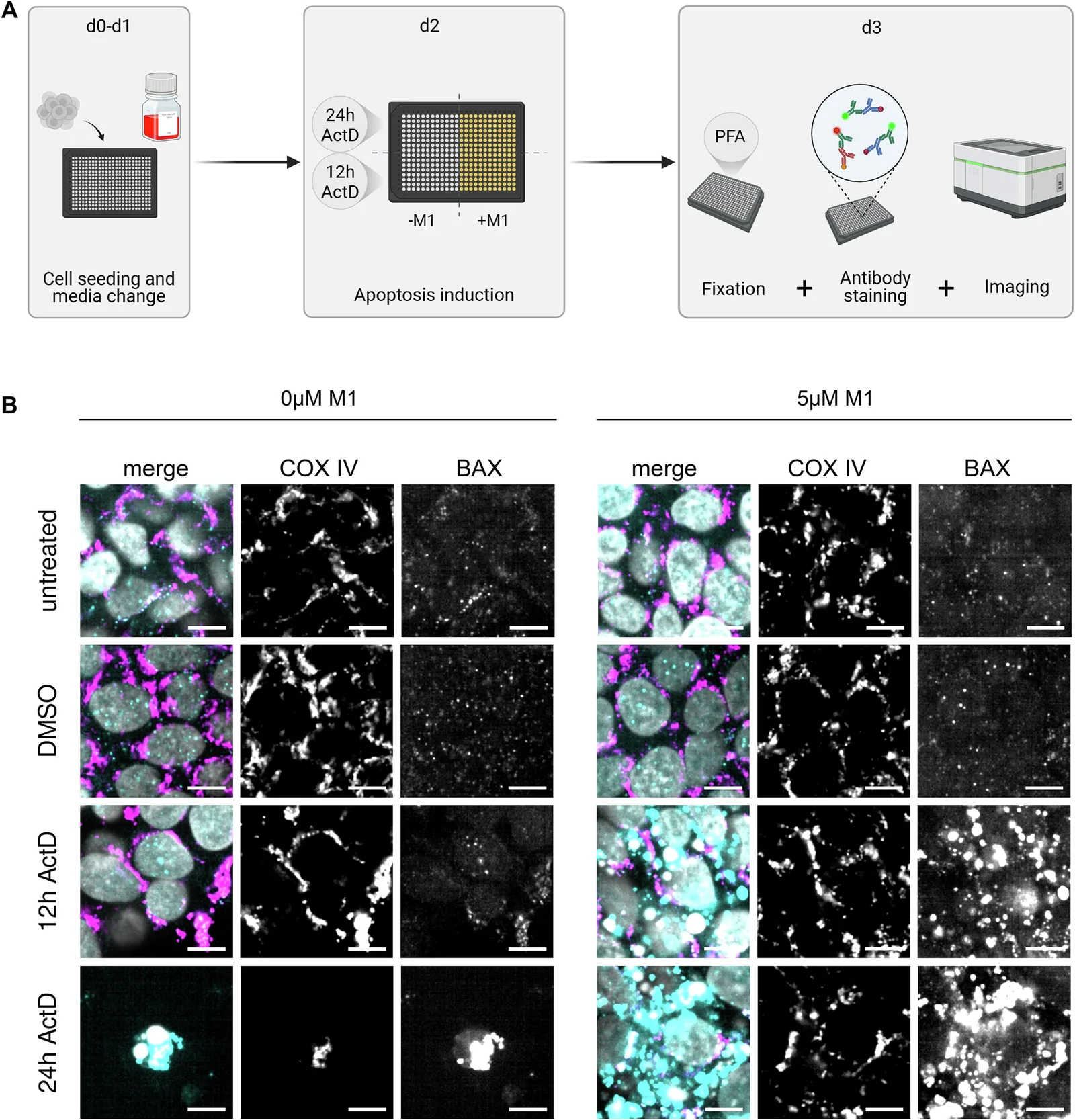
(**A**) Schematic overview of experimental setup. (**B**) Confocal images of hiPSCs with and without M1 and induction of apoptosis by ActD. “Untreated” refers to the absence of ActD treatment. Cells were stained with markers for mitochondria (COX IV, magenta), BAX (cyan), and nuclei (Hoechst, grey). COX IV and BAX are also shown as single channel images in grey scale. Scale bars: 10 µm. *Panel A was created with BioRender.com*.

Using Dataset 2 (Fig. 3), we examined how mitochondrial dynamics affect BAX accumulation in the MOM following an apoptotic stimulus in hiPSCs^21^. These cells were pre-treated with M1 or with DMSO as vehicle control for M1, and then exposed to 12.5 nM ActD or DMSO for 12 or 24 hours. Concentration and treatment duration for ActD were determined in pilot studies, with the intent of identifying conditions that reliably initiate apoptosis, as evidenced by BAX recruitment, while preserving cell attachment and permitting imaging. After fixation, we performed immunofluorescence staining for BAX and the mitochondrial inner membrane cytochrome c oxidase subunit IV (COX IV), which served as a structural reference for mitochondrial localization (Fig. 3). BAX recruitment to mitochondria assessed by immunofluorescence requires a fixed-cell, endpoint design for the direct spatial detection of endogenous BAX. This dataset thereby extends the observations made in mouse cells in Dataset 1 to a human, non-transformed cellular system. Because BAX redistribution is a critical event in the mitochondrial apoptotic pathway, this dataset offers a resource for investigating how the mitochondrial network state relates to BAX localization patterns at the single-cell level. To our knowledge, no publicly available immunofluorescence datasets currently combine BAX localization with a mitochondrial structural reference in hiPSCs under these experimental conditions.

## Methods

### Animal Ethics

Work with mouse embryos to generate MEF cultures was performed following local regulatory standards after approval by the regulating authority (Landesamt für Gesundheit und Soziales Berlin) under approval number T0123/16.

### Cell Culture

MEF cultures were generated based on established protocols^24^. Animal handling followed the PREPARE guidelines^25^. Briefly, pregnant female C57BL/6 J mice were euthanized by isoflurane overdose and E13 to E14 mouse embryos were harvested, euthanized by decapitation, washed in PBS, and organs (liver, intestines, lungs, heart, kidneys) were removed. The head was discarded. The remaining tissue was minced in ice-cold 0.05% Trypsin-EDTA (TE) and incubated at 4 °C overnight. After a subsequent 30-minute incubation at 37 °C, the tissue was triturated into a single-cell suspension, passed through a 70 µm cell strainer, and plated at 2*10^7^ cells per 175 cm^2^ flask. Cells were maintained in Dulbecco’s Modified Eagle’s Medium (DMEM, Thermo Fisher Scientific, 4.5 g/l glucose) supplemented with 10% foetal bovine serum (FBS), 1% L-glutamine (Thermo Fisher Scientific), 0.1% sodium pyruvate (Thermo Fisher Scientific), and 1% penicillin/streptomycin. MEFs were immortalized by long-term passaging and cultured in Dulbecco’s Modified Eagle’s Medium (DMEM) supplemented with 5% FBS. MEFs were cultured in a cell culture vessel until confluency reached about 80–90%, when cells were passaged using 0.25% trypsin/EDTA (Thermo Fisher Scientific, 25200056). Cells were cultured at 37 °C and 5% CO_2_ at all times.

Human induced pluripotent stem cell (hiPSC) line BIHi250-A (https://hpscreg.eu/cell-line/BIHi250-A) was grown on Geltrex (Thermo Fisher Scientific, A1413302)-coated cell culture vessels in Essential 8 (E8) media prepared based on the original recipe^26^. Media changes were performed daily for 5 days with one double-feed on day 6. When the confluency reached 75–80%, hiPSCs were passaged using 0.5 mM EDTA (Thermo Fisher Scientific, 15575020). HiPSCs were cultured at 37 °C and 5% CO_2_ at all times.

For all cell culture experiments, experiments performed from separate cell seedings were considered biological replicates (i.e. independent “N”).

### Apoptosis Assay on MEFs with Mitochondrial Dynamics Inhibitor

For apoptosis induction in MEFs, cells were seeded in 384-well µClear imaging plates (Greiner, 781091) at a density of 24,000 cells/cm^2^ and media was changed 24 hours later. The stock-solution (50 mM) of the mitochondrial dynamics inhibitor M1 (Sigma-Aldrich Merck, SML0629) was prepared in DMSO and sonicated at 40 kHz for 5 mins at room temperature (RT) before adding it to the respective wells in a 1 in 10,000 dilution to reach a final concentration of 5 µM. After 30 minutes of pre-treatment with M1 or its vehicle control DMSO, cells were exposed to actinomycin D (ActD, Selleckchem, S8964) in concentrations ranging from 0 to 120 nM. The “0 nM ActD” condition is referred to as “DMSO” in Fig. 2 and as “DMSO” or “M1 + DMSO” in Table 1. For the following live-cell imaging, tetramethylrhodamine-ethylester (TMRE, Thermo Fisher Scientific, T669) was added at a concentration of 50 nM followed by Annexin V-FITC (ImmunoTools, 31490013X2) according to the manufacturer’s instructions. It should be noted that the intensity of TMRE is mitochondrial membrane potential-dependent. This feature might be desirable in certain analytical scenarios, but might interfere with structural analyses in other cases.

**Fig. 4:**
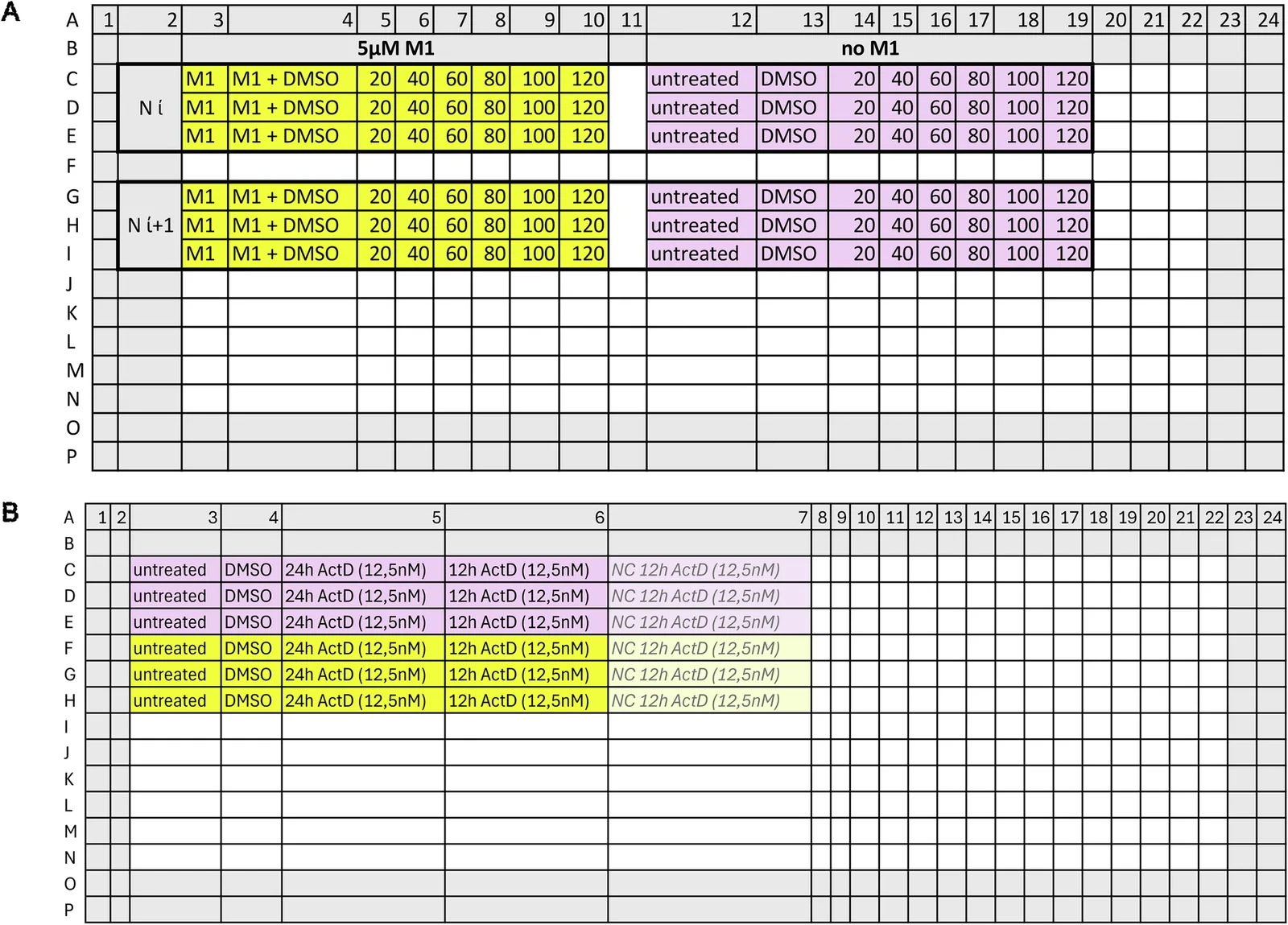
(**A**) Example plate layout for live-cell imaging experiments performed on MEFs. Wells shaded in yellow were pre-treated with 5 µM M1 for 30 minutes prior to apoptosis induction with ActD, while wells shaded in purple received DMSO as a vehicle control. Numbers within each well indicate the ActD concentration applied and wells displaying no number received 0 nM ActD but were otherwise treated as indicated. Wells labelled “N i” and “N i + 1” represent cells from two independent cultures constituting separate biological replicates. Note that plate layouts were varied across imaging sets to minimize potential edge effects associated with well position on the plate. Exact well indicators for treatments in all imaging sets can be found in associated file lists in the repository. (**B**) Example plate layout for immunofluorescence imaging performed on hiPSCs. Wells shaded in yellow were pre-treated with 5 µM M1 for 30 minutes prior to apoptosis induction with ActD, while wells shaded in purple received DMSO as vehicle control. Wells displayed in transparent shading served as no-primary antibody controls (NC), receiving all secondary antibody incubation steps but no primary antibody. Cells were treated as indicated in individual wells.

For this dataset, a total of 11 biological replicates were performed, using cells of different passages to ensure independent sampling. To increase experimental time efficiency, two biological replicates were conducted in parallel on the same plate, with each replicate occupying separate wells (see Fig. 4A for an example of the plate layout). In the first experiment (N1), only a single biological replicate was performed. For each biological replicate, every experimental condition was represented by three technical well-replicates. Information on experimental metadata can be found in Table 1.

### Live Cell Image Acquisition of MEFs

Live-cell imaging was conducted using a spinning-disk confocal microscope (Opera Phenix, Revvity) equipped with a 40× water immersion objective (NA = 1.1) and controlled by Harmony 5.2 software (Revvity). The microscope environment was set to 37 °C and 5% CO_2_ prior to the first imaging timepoint. Conditions were maintained throughout imaging time. TMRE fluorescence was excited using a 561 nm, 100 mW laser (emission filter: 570–630 nm) at 100% laser power and 80 ms camera exposure time. Annexin V-FITC was excited with a 488 nm, 150 mW laser (emission filter: 500–550 nm) at 50% laser power and 40 ms camera exposure time. DPC images were generated from two brightfield images (LED light source, 80 mW), acquired at −3 µm (lower plane) and + 5 µm (upper plane) relative to the focal plane. Both planes were illuminated for 700 ms at an illumination power of 50%. The default parameters for filter parameter (1.0) and speckle scale (10 µm) were kept for the measurement. Channels were acquired sequentially, with DPC imaged first, followed by TMRE and then Annexin V-FITC. Two image sequences were established for consecutive acquisition: during the first 10 h of imaging, images were recorded every two hours. Then, images were recorded every hour until the last acquisition after 40 h in total. For each well, 12 out of 81 possible fields of view for the entire well imaged with a 40× objective were predefined for image acquisition. Information on imaging metadata can be found in Table 1.

### BAX Immunofluorescence in hiPSCs

HiPSCs were dissociated with TrypLE Select (Thermo Fisher Scientific, 12563011) and seeded as single cells (24,000 cells/cm^2^) onto Geltrex-coated µClear 384-well plates (Greiner, 781091) in E8 medium containing 5 µM ROCK inhibitor (Y-27632, Selleckchem, S1049). After 24 h, the medium was replaced to remove the ROCK inhibitor, and cells were cultured for another 24 h prior to treatment with 12.5 nM ActD or its vehicle control DMSO, with separate wells treated for either 12 h or 24 h. Where indicated, cells were pre-treated with M1 or its vehicle control DMSO for 30 min before addition of ActD following the same preparation steps as described for MEFs above. At the experimental endpoint, cells were fixed with 4% paraformaldehyde (PFA, Carl Roth, 0964) for 15 min at 37 °C and rinsed twice with PBS without calcium or magnesium (PBS^−/−^) at RT. Blocking and permeabilization were carried out for 45 min at RT using 5% normal donkey serum (NDS, Merck Millipore, S30-100ML) and 0.3% saponin (Sigma-Aldrich Merck, 47036) in PBS^−/−^. Cells were then incubated for 1 h at RT with primary antibodies against COX IV (mouse mAB, 4D11-B3-E8, Cell Signaling Technology, 11967; 1:500) and BAX (rabbit mAb, D2E11, Cell Signaling Technology, 5023; 1:200) diluted in blocking buffer. After three 5-min washes in PBS^−/−^ at RT, secondary antibodies (donkey anti-mouse (HCA)-Alexa 647, Thermo Fisher Scientific, A31571; donkey anti-rabbit (HCA)-Alexa 488, Thermo Fisher Scientific, A32790) were applied at 1:500 in blocking buffer for 2 h at RT in the dark. Following three additional 5-min PBS^−/−^ washes, Hoechst 33342 (10 µM, Thermo Fisher Scientific, H1399) was included in the final wash. Samples were protected from light until imaging.

For this dataset, three biological replicates were performed independently using cells from different passages. For each biological replicate, three technical well-replicates per experimental condition were performed. Information on experimental metadata can be found in Table 2.

**Table 2:** Overview of Immunofluorescence Imaging of hiPSCs (Dataset2).

Imaging Set	Replicate ID	Cell Type	Passage Range	Treatment condition	ActD Conc [nM]	M1 Conc [µM]	Comment	No. of Wells	No. of Fields per Well	No. of Timepoints	No. of Planes	Channels	Objective	Image Size	Pixel Size [µm]	Bit Depth	Total Image Count
Immunofluorescence Imaging Set1	N1	hiPSC	8	Untreated	0	0		3	12	1	1	Hoechst, Cox IV, Bax	63x Water, NA = 1,15	1080x1080	0.19	16-bit	864
				DMSO	0	0	Diluent Control	3	12	1	1						
				24h ActD	12.5	0		3	12	1	1						
				12h ActD	12.5	0		3	12	1	1						
				M1	0	5		3	12	1	1						
				M1+DMSO	0	5	Diluent Control	3	12	1	1						
				M1+24h ActD	12.5	5		3	12	1	1						
				M1+12h ActD	12.5	5		3	12	1	1						
Immunofluorescence Imaging Set2	N2	hiPSC	10	Untreated	0	0		3	48	1	1	Hoechst, Cox IV, Bax	63x Water, NA = 1,15	1080x1080	0.19	16-bit	4320
				DMSO	0	0	Diluent Control	3	48	1	1						
				24h ActD	12.5	0		3	48	1	1						
				12h ActD	12.5	0		3	48	1	1						
				12h ActD	12.5	0	No primary antibody control	3	48	1	1						
				M1	0	5		3	48	1	1						
				M1+DMSO	0	5	Diluent Control	3	48	1	1						
				M1+24h ActD	12.5	5		3	48	1	1						
				M1+12h ActD	12.5	5		3	48	1	1						
				M1+12h ActD	12.5	5	No primary antibody control	3	48	1	1						
Immunofluorescence Imaging Set3	N2	hiPSC	11	Untreated	0	0		3	48	1	1	Hoechst, Cox IV, Bax	63x Water, NA = 1,15	1080x1080	0.19	16-bit	4320
				DMSO	0	0	Diluent Control	3	48	1	1						
				24h ActD	12.5	0		3	48	1	1						
				12h ActD	12.5	0		3	48	1	1						
				12h ActD	12.5	0	No primary antibody control	3	48	1	1						
				M1	0	5		3	48	1	1						
				M1+DMSO	0	5	Diluent Control	3	48	1	1						
				M1+24h ActD	12.5	5		3	48	1	1						
				M1+12h ActD	12.5	5		3	48	1	1						
				M1+12h ActD	12.5	5	No primary antibody control	3	48	1	1						

### Image Acquisition of hiPSCs

Imaging of hiPSCs with immunofluorescent staining for BAX and COX IV was conducted using a spinning-disk confocal microscope (Opera Phenix, Revvity) equipped with a 63x water immersion objective (NA = 1.15) and controlled by Harmony 5.2 software (Revvity). Hoechst was excited with a 375 nm, 50 mW laser (emission filter: 435–480 nm) at 40% laser power and 20 ms camera exposure time. BAX fluorescence was excited using the 488 nm laser (150 mW, emission filter: 500–550 nm) at a laser power of 80% and 80 ms camera exposure time. COX IV was imaged using a 640 nm, 150 mW laser (emission filter: 650–760 nm) at 80% power and 80 ms camera exposure time. All channels were acquired at an image height of 2 µm above the plate bottom. Hoechst and COX IV were recorded simultaneously, followed by a separate acquisition for BAX. A pixel binning of 2 × 2 was applied during acquisition to improve signal-to-noise ratio of fluorescence detection and considering varying signal intensity across samples. For the first imaging set, 12 predefined fields of view were captured per well. To improve cellular coverage, this was increased to 48 fields of view of 225 possible fields of view for the entire well imaged with a 63x objective for imaging sets 2 and 3 (see Fig. 4B for an example of the plate layout). Information on imaging metadata can be found in Table 2.

## Data Records

All images were exported from Harmony 5.2 (Revvity) as TIFF files, with file names automatically generated by the software using regular expressions. An example file name, r03c03f01p01-ch1sk1fk1fl1.tiff, is parsed as follows: row (r), column (c), field of view (f), z-plane (p), channel (ch), and timepoint (sk), where the number following each code indicates the respective index. The remaining abbreviations present in the file name (fk, fl) are further regular expressions that have no meaning in the current data set and remain constant across all exported files.

### Dataset 1 (Apoptosis in MEF)

This dataset contains confocal TIFF images of MEF cells stained with TMRE and Annexin V-FITC after exposure to the mitochondrial dynamics inhibitor M1 and varying concentrations of the apoptosis inducer ActD. Imaging sets 1 and 2 (i.e., biological replicates N1 – N3) contain images acquired at ActD concentrations 0, 20, 40, 60, and 80 nM, and imaging sets 3 to 6 (i.e., biological replicates N4 – N11) include ActD concentrations 0, 20, 40, 60, 80, 100, and 120 nM. Live-cell imaging was performed over 40 hours, as indicated above. Images were acquired in three channels: DPC (cell morphology), TMRE (mitochondria), and Annexin V-FITC (execution-phase apoptosis) (see Table 1).

The dataset contains six imaging sets with 11 biological replicates (N1 – N11; 46,656 to 124,416 files per imaging set). In total, the data set contains 637,632 images. For each experiment, a corresponding file list is included in the repository. These file lists document all file names, experimental conditions (ActD concentrations, M1 treatment, untreated), timepoints, and acquisition channels, and the complete file-naming scheme and directory structure. In addition, each imaging set includes an index.xml file located in the corresponding image folder on the BioImage Archive, consolidating plate-level, well-level, per-channel, and timepoint acquisition metadata for all images in that folder. Environmental monitoring data recorded during the acquisition are additionally embedded in the image header of each TIFF file.

The dataset and associated metadata files are available in BioImage Archive repository under https://www.ebi.ac.uk/biostudies/bioimages/studies/S-BIAD2490 (Ref. ^27^). We recommend consulting the help section of BioImage Archive for information on the different ways to download the data.

### Dataset 2 (Immunofluorescence Imaging of BAX)

This dataset comprises confocal TIFF images of hiPSCs stained by immunofluorescence for the proteins BAX and COX IV. Cells were treated with the mitochondrial dynamics inhibitor M1 and a single concentration of ActD for either 12 or 24 hours prior to fixation and staining for BAX, COX IV, and the nuclear stain Hoechst 33342. The dataset contains three biological replicates (N1: 864 files, N2 and N3: 4,320 individual files each), all generated using the same experimental design and acquisition settings. In total, the data set contains 9,504 images (see Table 2).

For each replicate, a corresponding file list is included in the repository. These file lists document all file names, experimental conditions (ActD exposure time, M1 treated / untreated), antibody names and dilutions, acquisition channels, and the complete file-naming scheme and directory structure. Furthermore, each image folder on the BioImage Archive also includes an index.xml file, which contains plate-level, well-level, and per-channel acquisition metadata for all images in that folder.

The dataset and associated metadata files are available in BioImage Archive repository under https://www.ebi.ac.uk/biostudies/bioimages/studies/S-BIAD2490 (Ref. ^27^). We recommend consulting the help section of BioImage Archive for information on the different ways to download the data.

## Technical Validation

### Instrument and data acquisition

All images of both datasets were acquired using the commercial Opera Phenix spinning-disk confocal microscope operated via the Harmony software v5.2 by Revvity. The microscope’s proprietary automated alignment function was used throughout all experiments including a channel-specific image alignment, which calculates and applies an image transformation per channel to correct for any image offset impairing image quality. Laser performance and filter sets were maintained according to the manufacturer’s recommended service schedule, ensuring stable excitation and emission properties throughout image acquisition. Images were recorded in the native Harmony file format and exported to TIFF using Harmony 5.2 to contain all relevant metadata.

### Cells

Cells were regularly monitored to be free of mycoplasma. HiPSCs were cultured according to standardized protocols and verified for stem cell identity and chromosomal integrity.

### Live-Cell Imaging

To ensure reliability, images of all live-cell imaging experiments were acquired with the same acquisition parameters such as laser intensities, exposure time, and focus height (see methods). Live-cell images were acquired with environmental conditions set to 5% CO_2_ and 37 °C controlled by the Harmony software. As baseline control, cells without ActD or M1 treatment were included to monitor technical variability and cell health during imaging. Because DMSO was used as solvent for ActD and M1, cells treated with DMSO only served as control to confirm that no artefacts were introduced by the vehicle. Additional wells containing cells treated with M1 alone served as further controls. Figures 1,2 show representative fluorescence images of the control conditions, with cellular morphologies consistent with the expected biological processes. Quantitative analyses performed as part of the underlying biological study indicated that M1 treatment alone did not introduce detectable changes in the imaging readouts specific for apoptosis used in this dataset^21^.

To confirm that the fluorescent signal was captured within the dynamic range of the detector, pixel intensity distributions were examined in representative images across the TMRE and Annexin V-FITC channels. For timepoint 1, intensity values for TMRE ranged from 173–24371 within the 16-bit dynamic range of 0–65535 and Annexin V-FITC ranged from 79–3555. Intensity values at the last timepoint ranged from 310–29347 (TMRE) and 79–22804 (Annexin V-FITC). This confirms that signal was captured well above background noise without evidence of pixel saturation and indicates appropriate acquisition settings across the dataset.

### Immunofluorescence Imaging

The immunofluorescence imaging dataset was acquired using consistent acquisition parameters across all replicates. For each experiment, cells were stained for BAX, COX IV, and Hoechst. In experiments 2 and 3 a subset of cells was included as no-primary-antibody control to assess potential background signal. In addition, untreated cells as well as cells treated with DMSO alone, the solvent for both ActD and M1, were included to confirm that no technical artefacts were introduced by the vehicle.

Pixel intensity distributions of representative images were examined across all channels to confirm that the fluorescence signal was captured within the dynamic range of the detector. Out of the full range of the 16-bit dynamic range (0–65535) the Hoechst 33342 channel ranged from 89–11356, COX IV signal ranged from 89–2561, and BAX signal ranged from 57–7363. Pixel intensity values across all channels remained within the dynamic range of the detector, with no evidence of signal saturation, and signal levels consistently exceeded background noise thresholds, supporting the suitability of the acquisition settings applied throughout this dataset.
